# Increasing Pump–Probe Signal toward Asymptotic Limits

**DOI:** 10.1021/acs.jpcb.3c01270

**Published:** 2023-05-18

**Authors:** Kevin C. Robben, Christopher M. Cheatum

**Affiliations:** Department of Chemistry, University of Iowa, Iowa City, Iowa 52242, USA

## Abstract

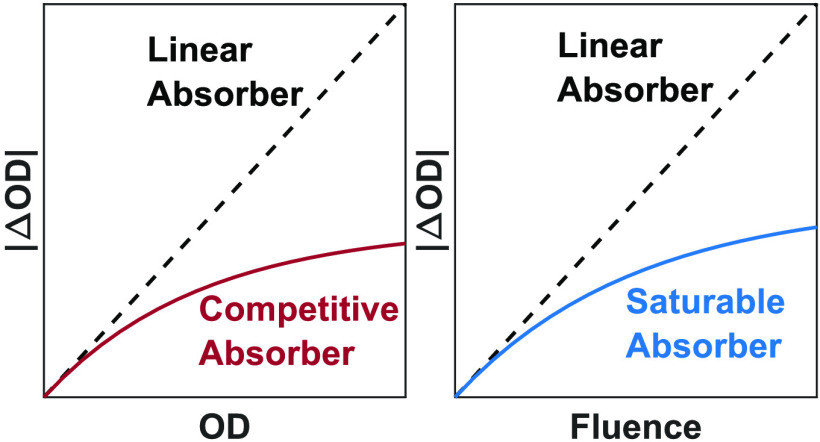

Optimization of pump–probe signal requires a complete understanding of how signal scales with experimental factors. In simple systems, signal scales quadratically with molar absorptivity, and linearly with fluence, concentration, and path length. In practice, scaling factors weaken beyond certain thresholds (e.g., OD > 0.1) due to asymptotic limits related to optical density, fluence and path length. While computational models can accurately account for subdued scaling, quantitative explanations often appear quite technical in the literature. This Perspective aims to present a simpler understanding of the subject with concise formulas for estimating absolute magnitudes of signal under both ordinary and asymptotic scaling conditions. This formulation may be more appealing for spectroscopists seeking rough estimates of signal or relative comparisons. We identify scaling dependencies of signal with respect to experimental parameters and discuss applications for improving signal under broad conditions. We also review other signal enhancement methods, such as local-oscillator attenuation and plasmonic enhancement, and discuss respective benefits and challenges regarding asymptotic limits that signal cannot exceed.

## Introduction

Ultrafast pump–probe spectroscopy is a powerful tool for measuring the structural dynamics and functions of molecules ranging between the millisecond and attosecond time scales.^1−3^ The technique consists of measuring the change in absorption of a probe pulse following a prior interaction with a pump pulse. Variations of the technique can differ by the number of independent variables resolved in a spectrum. In the simplest case, transient absorption spectroscopy resolves a spectrum as a function of the probe frequency and time delay between the pump and probe, enabling observation of how certain chemical species or populations change as a function of time. Transient absorption has seen a variety of applications, with examples ranging from photoinduced processes in molecular biology,^4−6^ polaritons in waveguides,^7^ and energy or charge transfer in nanostructures^8,9^ and photovoltaics.^10^

Time-resolved two-dimensional (2D) spectroscopy is another variation of the pump–probe technique that contains all the same information as transient absorption, while also adding more by resolving the spectrum as a function of both pump and probe frequencies. This enables disambiguation of combination bands, overtones, and Fermi resonances and allows for observing how populations evolve over time through correlations in frequency. Polarization schemes offer further insight into molecular structure through the relative orientation of modes.^11^ Applications of 2D spectroscopy are far reaching, with examples ranging from investigating protein folding motions,^12−14^ protein structure and dynamics,^15,16^ chemical reactions,^17,18^ solvent dynamics,^19,20^ mechanisms of charge separation and energy transfer in photosynthetic systems,^21−25^ and excitons in semiconductors.^26^

The improvement of signal-to-noise in pump–probe spectroscopy remains an ongoing challenge in order to expand the range of systems accessible by the technique.^27^ This requires a complete understanding of how signal scales with experimental factors. In the simplest case, signal scales quadratically with molar absorptivity and linearly with fluence, concentration, and path length. But conventional scaling laws do not account for asymptotic limits related to optical density, fluence, and path length. For example, the upper limit of signal for a two-level system under incoherent excitation is |ΔOD| ≤ OD, with equality corresponding to 100% transmission of the probe beam through the pumped sample. Furthermore, this upper limit is only valid for small optical densities (<0.1), but as we discuss later, increasing the optical density of a sample leads to higher attenuation of the pump beam and therefore weakens signal scaling factors. For example, increasing the optical density of a sample from 0.05 OD to 0.1 OD leads to a 1.9× increase in signal, but an increase from 0.5 OD to 1.0 OD merely leads to a 1.3× increase in signal. Accounting for solvent absorption and fluence further complicates scaling factors. Hence, conventional scaling laws are inadequate beyond a very limiting case of experimental conditions, and a more generalized formulation is necessary for accurately understanding how signal scales with experimental factors.

Early studies of photon echoes identified a nonlinear relationship between signal intensity and optical density due to accumulated attenuation of the excitation beams passing through the sample.^28,29^ Decades later, Jonas and co-workers explored the effect of optical density on signal both experimentally and theoretically through the use of beam propagation models based upon Maxwell’s equations^30−32^ or a modified form of Beer’s law to account for third-order response.^33,34^ In addition to high optical density, early studies of photon echoes also identified a nonlinear relationship between signal and intensity (or fluence).^35^ For a two-level system, the ratio of ground state to excited state oscillators cannot exceed unity due to competing processes of absorption and stimulated emission, which is the basis for fluence saturation.

Many of the formulas here build on previous work,^36^ including an approximation to another solution published by Cho et al.^33^ A number of publications have reported formulas for estimating relative quantities of signal, concentration, and cross sections but require a secondary measurement or calibrant to compute absolute quantities.^34,37−40^ Here we identify concise formulas for calculating absolute values of signal in cases of high optical density and/or high fluence given known quantities of molar absorptivity, concentration, path length, pulse energy, spectral bandwidth, line width, beam waist at focus, and optical density of the solvent. Furthermore, we analyze these formulas in the context of experimental strategies to enhance signal and identify asymptotic limits that signal cannot exceeded.

Following is the layout of the manuscript: Section 1 provides a quick review of Gaussian beams necessary for later formulas. Section 2 derives a model for optically dense samples while section 3 derives a model for high fluence measurements, and Section 4 derives a model suitable in both limits. Section 5 analyzes these formulas to identify trends for improving signal and recognizing asymptotic limits. Sections 6 and 7 review additional forms of signal enhancement including local oscillator attenuation and plasmonic enhancement, with discussion of how scaling laws and asymptotic limits apply in each case. Finally, Section 8 reviews a close relationship between temperature jumps and signal that is particularly relevant to aqueous samples excited in the infrared.

## 1 Gaussian Beams

**Figure 1:**
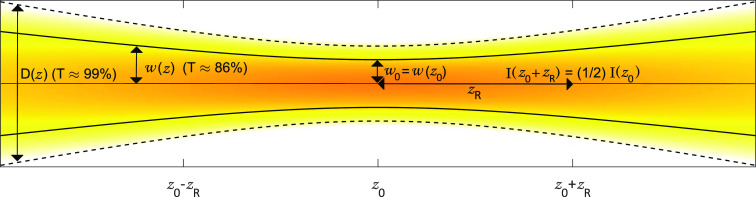
Characteristic measures of a Gaussian beam, including spot size , beam waist , Rayleigh length , and aperture diameter .

The Gaussian beam is a solution to the scalar wave equation that assumes a laser beam primarily propagating in one direction () and that the envelope slowly varies along . An important feature of Gaussian beams is that a point of focus must exist somewhere along the axis of propagation. This captures the reality that infinitely collimated beams do not exist. Rather, well-collimated beams are slowly converging, or diverging, to, or from, a point of focus. Figure 1 illustrates a few common measures of Gaussian beams. Siegman defines the *spot size* as the radius of aperture that transmits ∼86% of the beam’s power.^41^Equation 1 provides the formula for calculating the spot size in terms of the *beam waist* located at the focus , and the *Rayleigh length* at which the on-axis intensity of the beam is 1/2 the intensity at the focus, or equivalently, where . And finally, sometimes it may be convenient to reference the *aperture diameter* that corresponds to ∼99% transmission of the beam’s power. Note that Figure 1 is an equally valid model for both focused and collimated laser beams.
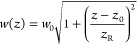
1

## 2 Competitive Absorber

**Figure 2:**
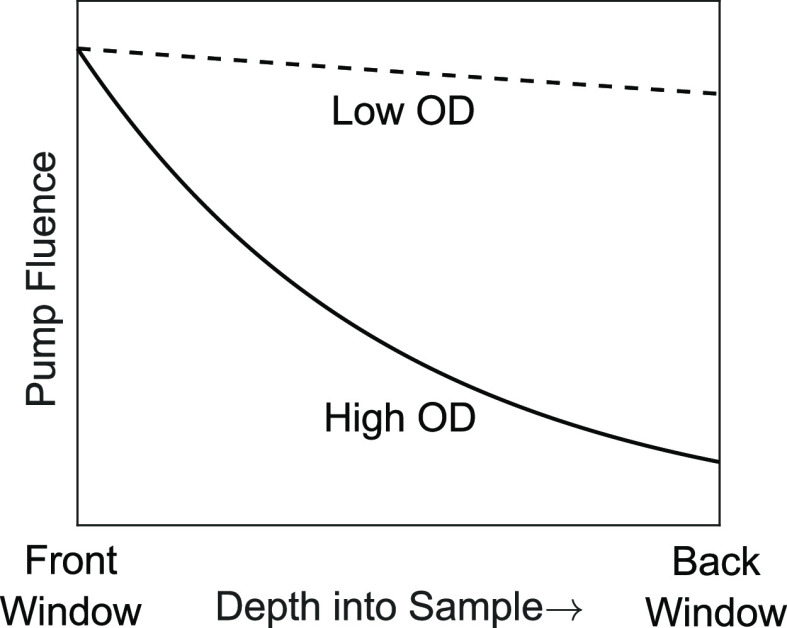
Plot of pump fluence as a function of the propagation depth into the sample in cases of low and high optical density.

The simplest model for pump–probe signal assumes scaling of ΔOD ∝ *F*ε_a_^2^*c*_a_, where *F* is the fluence (energy/area) of the pump beam, ε_a_ and *c*_a_ are the molar absorptivity and concentration of the analyte, and  is the path length of the sample. One shortcoming of this model is the assumption that pump fluence stays constant as the beam passes through the sample. As illustrated in Figure 2, this assumption is valid in low OD samples (OD < 0.1) but inaccurate in higher OD samples where increasing absorption of the pump beam leads to subdued scaling in the signal. This effect is well-known and captured in models by Jonas and co-workers but requires numeric integration.^33^ Here we present a shortened version of their model based on Beer’s law that utilizes integral approximations to generate a concise formula. For simplicity’s sake, our derivation assumes a simple two-level system. We also note that similar shortened formulas have recently appeared in literature.^36,40^

We define signal, ΔOD, as the change in optical density of the probe pulse between pump “on” and “off”, defined in eq 2. The pump off optical density is simply equal to . The pump on optical density is equal to  – , where the first term accounts for absorption from the pump depleted ground state and the second term accounts for stimulated emission from the pump induced excited state. Hence, the net change in optical density is equal to eq 3. One may multiply eq 3 by additional terms to account for auxiliary effects like polarization, lifetime decay and rotational diffusion.^11,33,38^
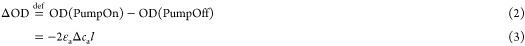


The factor of Δ*c*_a_ in eq 3 is equal to the number of pump photons absorbed by the sample divided by the volume of excitation. In the low fluence limit, for a nonabsorbing solvent, the total number of photons absorbed by the analyte is Δ*N*_abs_ = *N*_res_(1 – 10^–OD^), where *N*_res_ is the number of incident photons *resonant* (overlapping) with the oscillator’s line shape. For an absorbing solvent, Δ*N*_abs_ is reduced by a factor of  where OD_a_ and OD_s_ are optical densities of the analyte and solvent and OD_T_ is the sum total of the two. Hence, the number of photons absorbed by the analyte is . We approximate the observation volume as a cylinder with radius  and path length : . Equation 4 combines Δ*N*_abs_ and *V*_obs_ to estimate the average concentration of analyte excited by the pump beam. Division by Avogadro’s number *N*_A_ in eq 4 ensures Δ*c*_a_ carries units of molarity.

To calculate *N*_res_, we assume the pump spectrum and oscillator line shape are both Gaussian, with FWHM denoted by Δ_bw_ and Δ_lw_, respectively. Furthermore, we assume that Δ_bw_ ≫ Δ_lw_, which enables the approximation , where *N*_tot_ is the total number of pump photons. *N*_tot_ is related to the pump pulse energy by *N*_tot_ = *E*_pump_/*hc*ν̃, where *h* is Planck’s constant, *c* is the speed of light and ν̃ is the center frequency of the pump spectrum. These expressions combine in eq 5 to estimate *N*_res_.
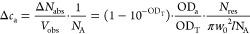
4
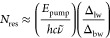
5

We recast ΔOD into eq 6a based on substitutions of Δ*c*_a_ from eq 4 and *N*_res_ from eq 5. The formula is quite simple when factored in terms of the saturation fluence; hence we have split a factor of ln(10)/ln(10) between eq 6a and eq 6c. The formula says that ΔOD of the analyte is equal to the product of two numbers. The first number, , is effectively the optical density of the analyte but adjusted for competing absorption of the pump light. Notice how  as OD_T_ → 0. The term “competitive” refers to any scenario in which OD_T_ is significant due to competing absorption by the solvent (OD_s_), the analyte (OD_a_), or both. The second number, *F̃*/*F*_sat_, is a measure of saturation induced by the pump beam. In fact, the quantity approximates twice the relative change in population, *F̃*/*F*_sat_ ≈ 2Δ*c*_a_/*c*_a_, in the low fluence and low OD limit.
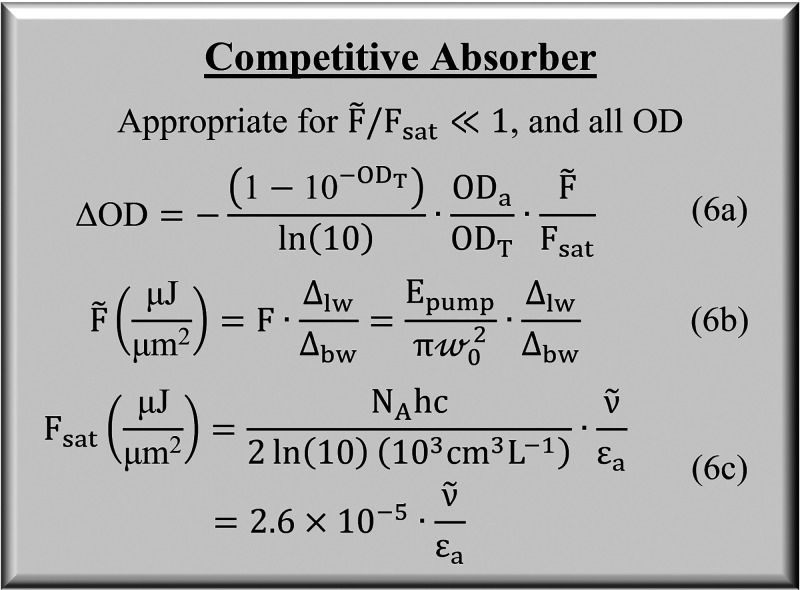
6

**Figure 3:**
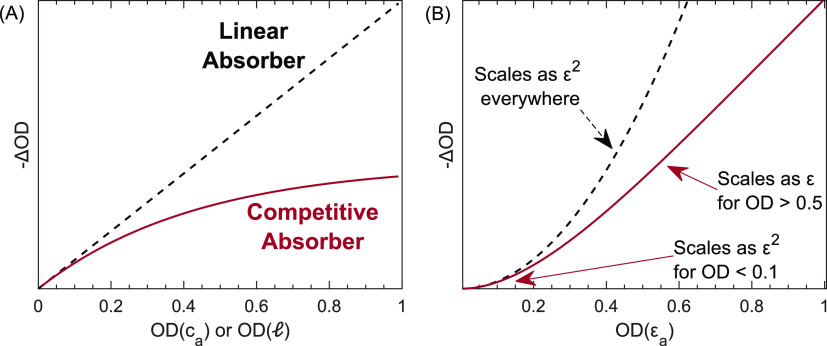
Comparison of ΔOD scaling for linear and competitive absorbers. (A) Scaling with respect to OD as a function of concentration or path length. (B) Scaling with respect to OD as a function of analyte molar absorptivity.

We refer to the conventional scaling law, ΔOD ∝ *F*ε_a_OD, as “linear” because it assumes ΔOD is bilinear in optical density and fluence for constant ε_a_. Figure 3 compares signal scaling between the linear and competitive absorber models. In each panel, we plot ΔOD vs OD while varying OD as a function of *c*_a_, , or ε_a_. As shown in panel A, with respect to changes in concentration or path length, the two models are similar below OD < 0.1 and then diverge with increasing OD due to the asymptote imposed by competitive absorption of the pump light. This stems from the factor of (1 – 10^–OD^) in eq 6a that accounts for the depletion of the pump beam as it penetrates the sample, also illustrated in Figure 2. Above OD > 0.5, the sample has absorbed >68% of the pump light and attempting to increase ΔOD by increasing path length or concentration of the analyte returns dwindling gains. This trend agrees with prior observations in the literature.^33,40,42^ The dependency on ε_a_ is similar to a higher order. Because eq 6a scales as (1 – 10^–ε_a_^)ε_a_, ΔOD(ε_a_) transitions between quadratic scaling below OD_a_ < 0.1 to linear scaling above OD_a_ > 0.5. Due to the impact of OD on signal scaling, we divide our discussion in section 5 into categories of weak (OD < 0.1) and strong (OD > 0.5) absorption.

**Figure 4:**
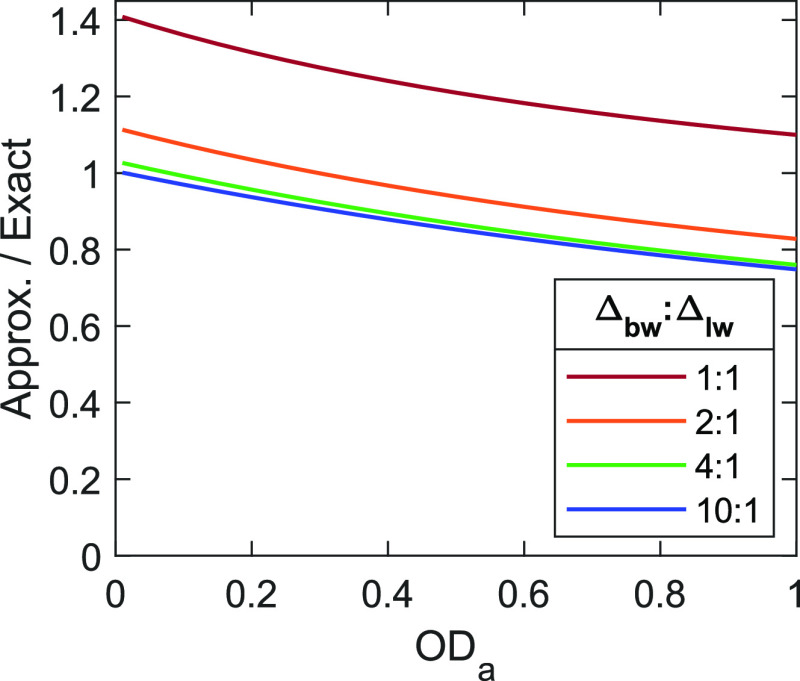
Comparison of ΔOD for the competitive absorber model computed by approximate and exact solutions to Beer’s law, as a function of optical density, for varying ratios of bandwidth to line width.

Figure 4 compares ΔOD between the competitive absorber model in eq 6a, which is an approximate solution to Beer’s law, to the exact solution derived by Jonas and co-workers, which models experimental results with high accuracy.^33^ These results assume a two-level system in the low fluence limit, pumped and probed by identically sized Gaussian beams. We find strong agreement between the competitive absorber model and the Jonas model (>90%) for systems with OD_a_ < 0.3 measured by large spectral bandwidths (2× line width or greater), and we still find good agreement (>60%) in more extreme cases. The agreement between solutions is explainable through integral approximations. The exact solution to Beer’s law is ΔOD = −log_10_(*P*_r_^on^/*P*_r_^off^) where *P*_r_^on^ and *P*_r_^off^ correspond to eqs 9 and 10 in ref (33), integrated across *X* and *Y* and evaluated at *Z* = . First, we approximate the pump and probe beams as averaged flat top beams, which eliminates all *X* and *Y* dependencies and simplifies ΔOD ≈ Δ*α* d*Z*/ln(10). Then we approximate the integral in eq A7, ref (33), as the area of a triangle such that Δα ≈ *p*_*u*_*α*^0^ exp(−α^0^*Z*)2σΔ_bw_ evaluated at the line shape peak, and a factor of 2 accounts for GSB + SE. And, finally, the substitutions  and *F*_sat_ = *hc*ν̃/2σ and integration over *Z* yields the same expression as eq 6a. The limiting estimate here is the triangle approximation of the spectral overlap integral, which is valid at low OD_a_ and  ≳ 3, but as seen in Figure 4, is prone to underestimate absorption around the tails of the line shape at higher OD_a_ and overestimate when the bandwidth of the pump spectrum is comparable to the line width of the line shape.

## 3 Saturable Absorber

The competitive absorber model is valid at low fluence for any optical density. Now we discuss the saturable absorber model, which is valid at low optical density for any incoherent excitation fluence. At low intensities, chromophores act as “linear” absorbers which means that, given an infinitesimally thin sample, the number of photons absorbed by the sample is linearly proportional to the number of photons incident upon it, i.e., the Beer–Lambert law. The linear absorber model works well at low intensities, typical of an FTIR light source, where just a small fraction of oscillators (<1%) absorb light. But given a sufficiently intense beam of light, the Beer–Lambert law predicts a sample could absorb more photons than are oscillators in solution. Therefore, the linear absorber model is unphysical at high intensities. Alternatively, the saturable absorber model accounts for saturation effects due to finite population and converges to the linear absorber model in the low intensity limit.

The saturable absorber model has two limiting cases. One for continuous wave, where the lifetime of the oscillator mode is much longer than the duration of the light source, and the other for ultrashort pulses with durations much shorter than the lifetime of oscillator mode.^43^ The model yields slightly different formulas in each limit and work in different units: intensity (energy/area/time) for the continuous wave case and fluence (energy/area) for the ultrafast case. Assuming a femtosecond light source, we restrict our analysis to the ultrafast limit.

**Figure 5:**
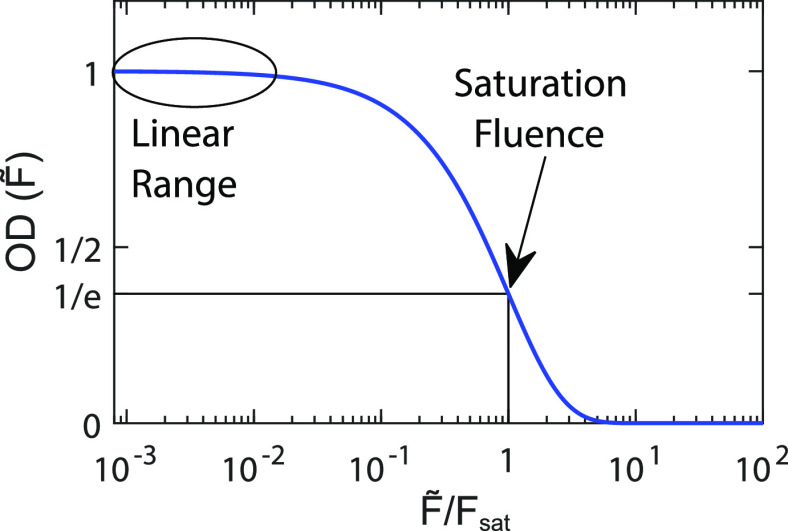
Plot of absorption versus resonant fluence for a saturable absorber measured by an ultrafast pulse.

The saturable absorption model asserts that optical density decays exponentially with fluence, as defined by eq 7a.^43^ As with the competitive absorber model before, only the resonant fluence, *F̃*, defined in eq 6b, is relevant here. We plot OD(*F̃*) versus *F̃*/*F*_sat_ in Figure 5. In the low fluence limit (*F̃*/*F*_sat_ ≲ 10^–2^), OD is constant, consistent with linear absorption, and then precipitously drops after *F̃*/*F*_sat_ ≈ 10^–1^ and approaches an asymptotic limit of OD = 0 above *F̃*/*F*_sat_ ≳ 5.

Equation 7b models ΔOD of a saturable absorber. In the high fluence limit, ΔOD → –OD, corresponding to 100% transmission of the pump sample. In the low fluence limit, all three models (competitive, saturable, and linear absorbers) converge to the same limit: ΔOD → −(*F̃*/*F*_sat_)OD. These formulas suggest a strong connection between saturable absorption and pump–probe signal. Indeed, for a two-level system, the change in population follows a saturation curve. Therefore, the two subjects are closely related. While this point appears to have been understood several decades ago,^44^ the connection between the two subjects has rarely surfaced within the ultrafast community since. We hope to revive this perspective given the remarkably simple formulation it provides for computing ΔOD. One limitation with this formulation is it does not account for subdued scaling effects in high OD samples due to competitive absorption.
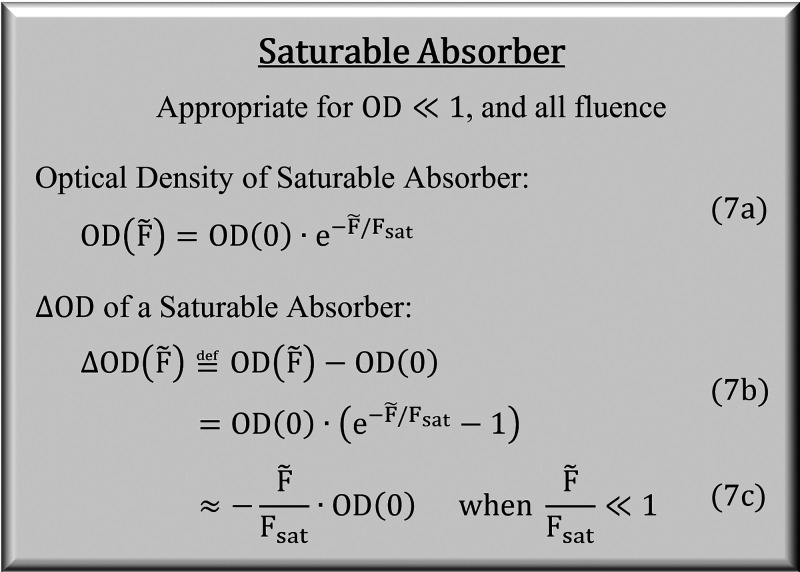
7

**Figure 6:**
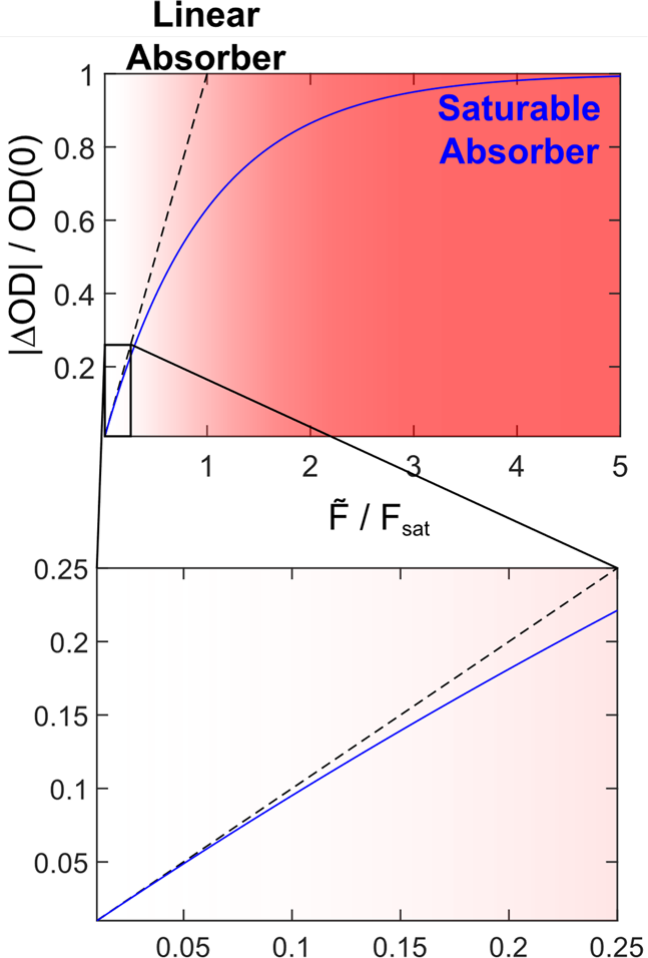
Pump–probe signal as a function of resonant fluence for a linear absorber and a saturable absorber. OD(0) is the linear absorbance measured in the weak fluence limit.

According to eq 7b, the ratio  is a useful measure of saturation. Figure 6 plots this ratio for both models as a function of *F̃*/*F*_sat_. Not surprisingly, |ΔOD|/OD(0) is linear in the weak fluence limit but appears increasingly nonlinear at higher fluences. Generally speaking,  ≳ 0.3 appears unlikely for multilevel systems because up-pumping will push population from ν = 1 into higher states to a point where |ΔOD| of the 0–1 will actually decrease with additional fluence.^45^ However, one way to avoid up-pumping is positively chirping the pump pulse.^46^ Alternatively, when a pulse shaper is available, applying a mask that removes pump light coincident with the 1–2 transition (e.g., a sufficiently narrow Gaussian mask centered at the 0–1 transition, or a hole centered at the 1–2 band) prevents up-pumping by disabling excitation to ν = 2 and higher.

Sampling a few examples from literature, data reported by both Witte et al. for W(CO)_6_ in a regular pump–probe experiment^45^ and Morichika et al. for plasmonic enhancement of W(CO)_6_ in reflection mode,^47^ both equate to a saturable limit of  ≲ 0.3, which is attributed to the up-pumping seen in both reports. Another convenient property of this ratio is that  should approximate the factional change in population, which appears confirmed by the data published by Witte et al.^45^ Another interesting example is azide in FDH, where  = 0.22, which implies a near-saturated response in these experiments.^48−50^ While the effects of saturation on measuring spectral diffusion have not been studied, for measurements in the (τ_1_, *T*_W_, ω_3_) domain, it is unlikely that a 10% nonlinearity is going to change spectral diffusion in any meaningful way.

These examples show that strong chromophores such as azide and metal carbonyls have either already or nearly reached a saturation point on standard 2D IR apparatuses and further increases in pump fluence are unlikely to improve . On the other hand, some room remains for fluence enhancement on weaker chromophores. For example, our recent reports with MeSCN (ε ≈ 150 M^–1^ cm^–1^) have ratios of  ≈ 0.01 and 0.05,^51,52^ which leaves room for ∼20× and ∼4× enhancement by increasing the pump fluence.

Finally, it is worth noting the existence of Rabi oscillations. Upon excitation by a coherent light source, a two-level system well-isolated from dephasing and relaxation processes will oscillate in population between the ground and excited state as a function of fluence. The frequency of oscillation, known as the Rabi frequency ω_R_, is proportional to the product of the electric field amplitude and the transition dipole moment. Although the saturable absorber model assumes an incoherent pump source, the model is still appropriate for coherent light sources when ω_R_ is smaller than the homogeneous line width because dephasing destroys the Rabi oscillation before it can significantly manifest.^41^

## 4 Saturable Competitive Absorber

The models reviewed up to this point are suitable for either high optical density or high fluence, but neither model is appropriate under both conditions. Here we derive a model suitable under both conditions which we call the saturable competitive absorber. The trick here is finding an expression that computes the change in population accounting for both saturation and high optical density. For the competitive absorber model, the change in ground state analyte from excitation by the pump beam at point *z* in the sample is Δ*c*_a_/*c*_a_ = (1/2)(*F̃*/*F*_sat_)10^–OD^ where *F̃* is the resonant fluence prior to entering the sample. This fails at high fluence because (*F̃*/*F*_sat_)10^–OD^ > 1 implies population inversion. To account for saturation, we replace the quantities *F̃*/*F*_sat_ and OD with their saturable counterparts from eqs 7a and 7b. Hence, Δ*c*_a_/*c*_a_ = (1/2)(1 – *f*)10^–*f* ·OD^, where *f* = exp(−*F̃*/*F*_sat_). This is consistent with the competitive absorber model in the low fluence limit, converging to (1/2)(*F̃*/*F*_*s*at_)10^–OD^, and the saturable absorber model in the high fluence limit, converging to 1/2. Then, following eq 3, we compute ΔOD by integrating Δ*c*_a_ = (*c*_a_/2)(1 – *f*)10^–*fc*_a_ε_a_*z*^ over *z* and multiplying by −2ε_a_, which gives eq 8a. This formula converges to all expected limits: −OD_a_ for *F̃*/*F*_sat_ ≫ 1, eq 6a for *F̃*/*F*_sat_ ≪ 1, eq 7b for OD_T_ ≪ 1, and eq 7c for *F̃*/*F*_sat_ ≪ 1 and OD_T_ ≪ 1.
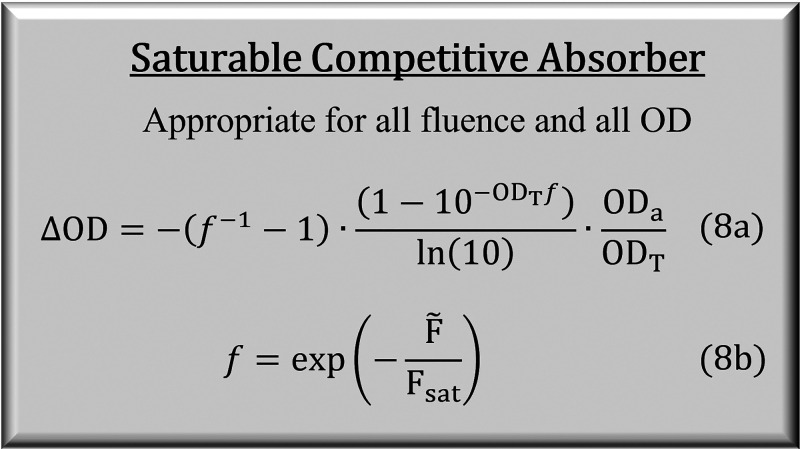
8

**Figure 7:**
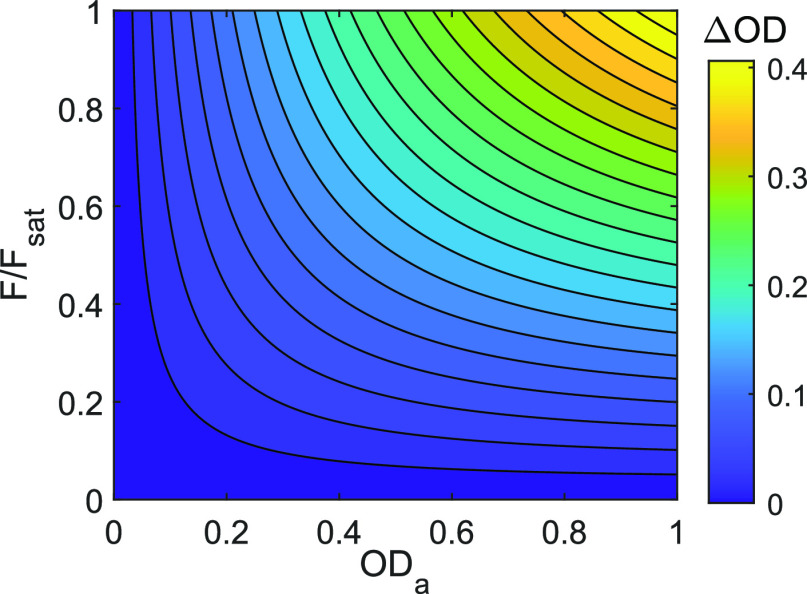
Contour plot of signal versus optical density and fluence for the saturable competitive absorber.

Figure 7 plots ΔOD as a function of optical density and fluence. The contours show that simultaneous increases in both optical density and fluence increase ΔOD, without an asymptote along the equidistant line. Higher fluence leads to more excitation, particularly toward the back end of high OD samples. Likewise, high optical density leads to more excitation. When possible, increasing both fluence and optical density could be a very potent strategy for improving signal, with the upper limit on signal only limited by the optical density of the sample. However, this could be problematic given line shape distortions are known to occur at high optical densities.^31,32^

## 5 Conventional Methods of Enhancement

Analysis of the competitive absorber model in eq 6a shows that the pump–probe signal depends on several different variables. We refer to conventional methods of enhancement as any strategy that targets these variables. Table 1 summarizes how ΔOD scales with respect to each of these variables for the two limiting cases of weak absorption (OD < 0.1) and strong absorption (OD > 0.5). The section reviews each variable dependence in detail and discusses practical and theoretical limits related to signal enhancement.

**Table 1:** Scaling Dependencies of the Pump-Probe Signal (ΔOD) for the Competitive Absorber Model in the Low Fluence Limit^a^

	Low OD (<0.1)^b^	High OD (>0.5)
Inverse Beam Waist (	**Quadratic**	Quadratic
Analyte Molar Absorptivity^(a)^	Quadratic	Linear
Pump Energy (Resonant Bandwidth)	Linear	Linear
Concentration of Analyte^(a)^	Linear	Bounded-Exponential
Path Length^(T)^	**Linear**	Bounded-Exponential
Solvent Molar Absorptivity	Hyperbolic Decay	Hyperbolic Decay

a (a) Distinguishes limits of absorption relative to OD of analyte and (T) distinguishes limits of absorption relative to OD of analyte + solvent.

b Bold text highlights canceling effects between the beam waist and optimal path length in the limiting case of weak absorption (section 5.4).

### 5.1 Decrease the Beam Waist/Increase the Mode Size

Equation 6a shows that ΔOD scales as , and therefore decreasing the beam waist  at the focus increases the pump–probe signal quadratically. Assuming a Gaussian beam, Siegman approximates the beam waist radius as eq 9 where *f* is the focal length, λ is the center wavelength, and *D* is the diameter of aperture at which 99% of light transmits just prior to reaching the focusing optic.^41^ For example, using *f* = 50 mm parabolic mirrors, *D* = 20 mm irises, and λ = 5 μm light, eq 9 expects a beam waist radius of  ≈ 12.5 μm. According to eq 9, there are only two ways of decreasing the beam waist  for a given wavelength: either reduce the focal length or increase the diameter of the mode and aperture of optics. These two quantities often limit each other through what is known as the “*f*-number” of an optic.
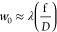
9
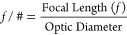
10The *f*-number (*f*/#), defined in eq 10, is equal to the focal length divided by the diameter of the focusing optic. Note that the diameter of the optic does not necessarily equal the aperture diameter *D* at which 99% of light transmits, but the smallest possible beam waist  does appear when the two quantities are equal. Assuming the two are equal, then according to eq 9, for a given wavelength, the only way to reduce the beam waist  is to find an optic with a smaller *f*-number. Several vendors sell *f*/1 off-axis parabolic mirrors and *f*/0.5 ZnSe aspherical lenses. However, faster optics are unlikely due to manufacturing challenges.

Regardless of the optic chosen, it is important to match the aperture diameter *D* with the optic’s diameter of aperture to achieve the smallest possible beam waist . We reiterate this point because, in our experience, the output beams from OPA’s may have angular beam spread which might require a telescope to collimate. Furthermore, as later discussed in section 5.3, all “collimated” beams eventually diverge after a finite distance which may be significant after a couple meters of propagation. On the other hand, if a beam reaches the focusing element with an aperture diameter *D* smaller than the optic diameter, then eq 9 says that the beam waist  is unnecessarily large.

Note that eq 6a assumes matching mode sizes between the pump and probe. Other factors that can inflate the beam waist larger than predicted by eq 9 include spherical aberrations, wavefront distortion, and anything that might affect the beam quality factor (*M*^2^).^41,53^ Spherical aberrations are eliminated by replacing spherical optics with aspherical lenses or parabolic mirrors. Nanometer deviations in optical surfaces imprint wavefront distortions on laser beams. Manufacturers characterize surface flatness using a monochromatic reference, e.g., λ/4, where λ is usually 633 nm. Common values for surface flatness range from λ/2 ≈ 300 nm to λ/10 ≈ 60 nm. It is important to recognize that wavefront errors accumulate over optics, e.g., 16 reflections off λ/2 mirrors add in quadrature to 300 nm ×  = 1.2 μm. Therefore, reducing the number of optics, choosing optics with high surface flatness, and keeping optics clean are simple strategies to mitigate wavefront distortion.

### 5.2 Choose an Analyte with a High Molar Absorptivity

Equation 6a shows that ΔOD scales quadratically with molar absorptivity ε_**a**_ in the weak absorption limit and linearly in the high absorption limit. In terms of general site-specific labels in proteins, the IR transparency window of water effectively limits the available chromophores to those listed in Table 2. See refs (2), (54), and (55) for further review on site-specific infrared labels. While azides are brighter than nitriles, their short vibrational lifetimes (∼1 ps) seriously limit their ability to report spectral diffusion.

**Table 2:** Site-Specific Infrared Labels Suitable for 2D IR Spectroscopy of Proteins, Including Molar Absorptivity ε_a_ and Vibrational Lifetime *T*_1_

Class	Unnatural Amino Acid	Isotopologue	Frequency (cm^–1^)^c^	ε_a_ (M^–1^ cm^–1^)	*T*_1_^d^ (ps)	Ref
nitriles	4-cyanophenylalanine	Phe-^12^C^14^N	**∼2237**	∼180–220	∼4.1	(56−58)
		Phe-^12^C^15^N	**∼2210**		**∼0.9–2.2**	(56), (59)
		Phe-^13^C^14^N	∼2184		∼3.4–5.0	(56), (59)
		Phe-^13^C^15^N^a^	∼2156		∼8–11	(56), (58)
	4-cyanoselenophenylalanine	Phe-Se^12^C^14^N^a^	∼2160	∼65	∼76	(59)
	cyanocysteine	Cys-^12^C^14^N	∼2150–2180	∼35–120	∼30–60	(60−62)
	5-cyanotryptophan	Trp-^12^C^14^N	**∼2210–2240**	∼160	**∼1.4**	(63), (64)
azides	azidohomoalanine	Ala-^14^N_3_	∼2100–2140	∼300–400	**∼1.0**	(65)
	4-azidophenylalanine	Phe-^14^N_3_^b^	∼2120–2130	∼500–620	**∼0.7**	(66), (67)
	4-azidomethylphenylalanine	Phe-CH_2_^14^N_3_^a^	∼2110	∼310–370	N/A	(67)

a Notably more expensive than other UAAs on this table and/or requires considerable synthesis.

b Photoreactive with UVC light.

c Bold entries: May require purging of CO_2_ to avoid chromatic dispersion.

d Bold entries: Short vibrational lifetime limits spectral diffusion measurements.

### 5.3 Increase the Pump Energy

**Figure 8:**
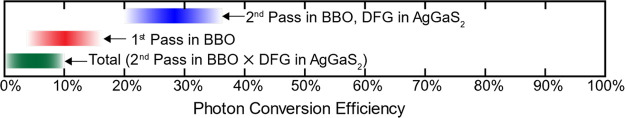
Typical photon conversion efficiency measured in near-IR BBO and mid-IR AgGaS_2_.

In the low fluence limit, eq 6a shows that ΔOD scales linearly with the energy per pump pulse, or more precisely, the spectral energy resonant with the analyte line shape. One approach to increasing pump energy is by improving the efficiency of mid-IR parametric amplifiers. An optical parametric amplifier works by splitting high frequency pump photons (ω_3_) into two lower energy photons, commonly referred to as the signal (ω_2_) and idler (ω_1_) where ω_3_ = ω_2_ + ω_1_.^68^ The photon conversion efficiency of an OPA, defined in eq 11, is the fraction of pump light split into lower energy fields. Given an input pump power *P*_3_(in) and input seed power much smaller than either the output signal power *P*_2_(out) or output idler power *P*_1_(out), the conversion efficiency is well-approximated by either of the latter two equalities in eq 11. Figure 8 illustrates typical photon conversion efficiency values for BBO and AgGaS_2_.^69^ Notice that power amplification stages, e.g., second pass in BBO or DFG in AgGaS_2_, tend to run upward of ∼30% while seed amplification stages, e.g., first pass in BBO, tend to run at ∼10%. Overall, the gross output power primarily comes from the power amplification stages (2nd pass in BBO and DFG in AgGaS_2_), so the total mid-IR photon conversion efficiency relative to the input Ti:sapphire pump is ∼(0.28)^2^ = 8%, which is typical in our experience (∼30 mW of mid-IR from ∼2200 mW of second pass pump). Beyond Ti:sapphire, some 2D IR groups have acquired 100 kHz Yb lasers with remarkable performance and applications.^70−75^
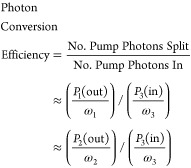
11

Note that ΔOD does not necessarily scale with the power but, rather, the energy or fluence per pump pulse. The distinction is significant from an SNR standpoint when comparing lasers with equal average power at different repetition rates. Consider, for example, a hypothetical comparison between a 1 kHz and a 100 kHz laser with identical average output powers. The 1 kHz laser yields a 100× larger signal owing to a larger pulse energy. On the other hand, the 100 kHz laser reduces noise first by a factor of 10× from  averaging, then by another factor of ∼10× from reduction in shot-to-shot fluctuations associated with 1/*f* noise. Overall, the two lasers should exhibit comparable values of SNR. In practice, the comparison is more complicated after factoring in specifications known for commercially available lasers, such as slightly higher average powers for 100 kHz systems, or implementation of calibrated referencing schemes known to remove virtually all shot-to-shot noise.^51,76,77^ And, finally, we note that only the fraction of the frequency spectrum that is resonant with the analyte line shape contributes to pump–probe signal, which is important when comparing two different light sources with different spectral bandwidths.

There are several sources of attenuation following the mid-IR light source, some of which are manageable. First is compounded reflection losses. For example, assuming gold mirror reflectivities of 98% for s-polarized light and 95% for p-polarized light, 15 reflections would accumulate 1 – 0.98^15^ = 26% power loss for s-polarized light and 1 – 0.96^15^ = 46% power loss for p-polarized light. Pulse shapers are known to attenuate pump light by 65–80%, depending on the circumstances. Taken altogether, for a typical 2D IR pulse shaping apparatus, one should expect the pump power at the sample to be 5–20% of the initial power measured at the mid-IR source.

The last source of attenuation worth noting is accidental divergence and/or long-range diffraction inherent to laser beams. Many 2D IR apparatuses propagate ∼5–8 m between the mid-IR source and sample cell. At these path lengths, small perturbations in the divergence angle might easily lead to major aperture losses in pump light. Furthermore, the diameter of the mode must be large enough to ensure the collimated range (twice the Rayleigh length) of the mode is long enough to reach the lens or parabolic mirror that focuses light into the sample. Equation 12 provides the formula for the collimated range of a Gaussian beam, borrowed from Siegman.^41^ Here λ is wavelength and *D* is the diameter of aperture for which 99% of light transmits at the two ends of the columnated range. For example, a 5 μm wavelength beam with diameter of aperture *D* = 10 mm can propagate 6 m before diverging larger than 10 mm again.
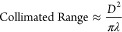
12

### 5.4 Increase the Analyte Concentration

Equation 6a shows that ΔOD scales linearly with *c*_a_ in the weak absorption limit or bounded-exponentially in the strong absorption limit. For proteins incorporating a single, site-specific residue as an analyte reporter, the absolute upper limit for concentration is equal to the inverse volume of a single monomer of the protein. By estimating the volume of a protein from its molecule mass and density,^78^ and ignoring excess volume occupied by water between protein molecules, the maximum concentration of a crystalline protein in units of molarity is approximately equal to 1.37 (M·kDa)/*m*(kDa), where *m* is the protein mass in units of kDa. If one then assumes the void between crystalline molecules is ∼1/3 of the crystal volume, then 1.37 becomes 1 and a very handy rule of thumb is that the maximum concentration of a crystalline protein in molarity is equal to the inverse mass in kDa. A typical protein monomer weighs anywhere from 10 to 50 kDa, which suggests an upper limit of *c*_a_ = 20–100 mM for crystalline protein. In practice, proteins start nucleating well before that point, which means the upper limit for a nonaggregate, solution phase measurement is somewhere between 0.1 and 10 mM, depending on the protein and experimental conditions.

### 5.5 Optimize the Path Length

**Figure 9:**
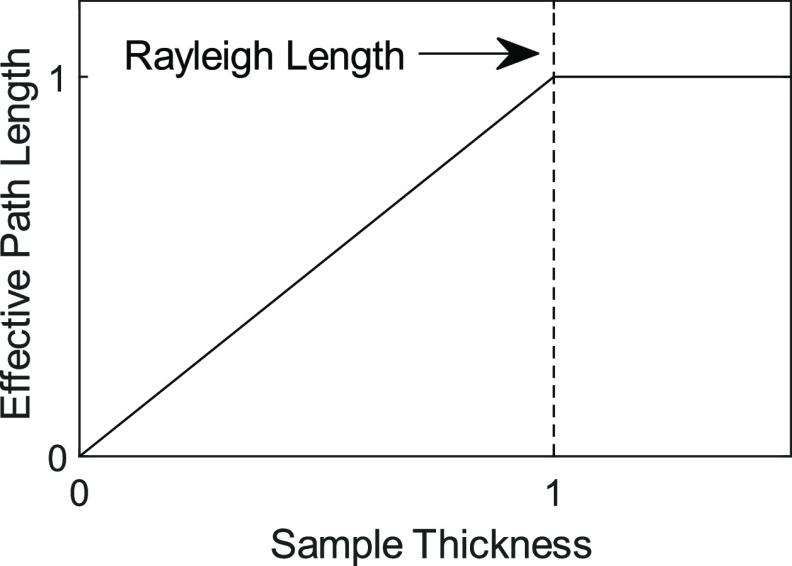
Plot illustrating the effective path length of the excitation volume relative to the Rayleigh length.

Equation 6a shows that ΔOD scales linearly with  in the weak absorption limit or bounded-exponentially in the strong absorption limit. However, this does not account for the overlap geometry. The Rayleigh length defines the distance at which the pump intensity falls to 1/2 the peak intensity, and therefore excitation of the analyte becomes negligible beyond a path length of . Furthermore, the geometry of overlapping pump and probe at a ∼30° angle shortens the observable path length of excited analyte by a factor of 2. Hence, the Rayleigh length  sets an upper limit signal dependency on path length (Figure 9). For convenience, eq 13 provides a formula for computing the optimal path length in terms of the beam waist and wavelength,^41^ or alternatively, in terms of the focal length *f* and aperture diameter *D* assuming *M*^2^ = 1. For example, given  = 12 μm and λ = 4.7 μm, eq 13 estimates a Rayleigh length of  ≈ 100 μm.
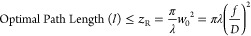
13

In practice, one may find that the optimal path length is significantly less than  depending on the optics, beam geometry and solvent. In the case of an absorbing solvent, one must consider the intensity of probe or local-oscillator light necessary to fill the well depth of the detector, which might further limit the path length. Interestingly, if we assume , then by eq 13. In the limit of weak absorption, Table 1 shows . Therefore, one should expect ΔOD does not depend on the beam waist when OD_T_ ≪ 1 and . In other words, given OD_T_ ≪ 1 and , ΔOD should not change with focusing assuming the path length is proportional to the Rayleigh length . Stated another way, ΔOD merely depends on the *energy* of the pump pulse rather than the physical dimensions of the observation volume, which is more intuitive. In practice, independence of ΔOD on the beam waist is unlikely merely because most samples have significant absorption from either the analyte or solvent. In moderate to strongly absorbing samples, the dependence of ΔOD on the inverse beam waist should be sublinear assuming an optimized path length.

### 5.6 Choose a Solvent with a Low Absorptivity

**Figure 10:**
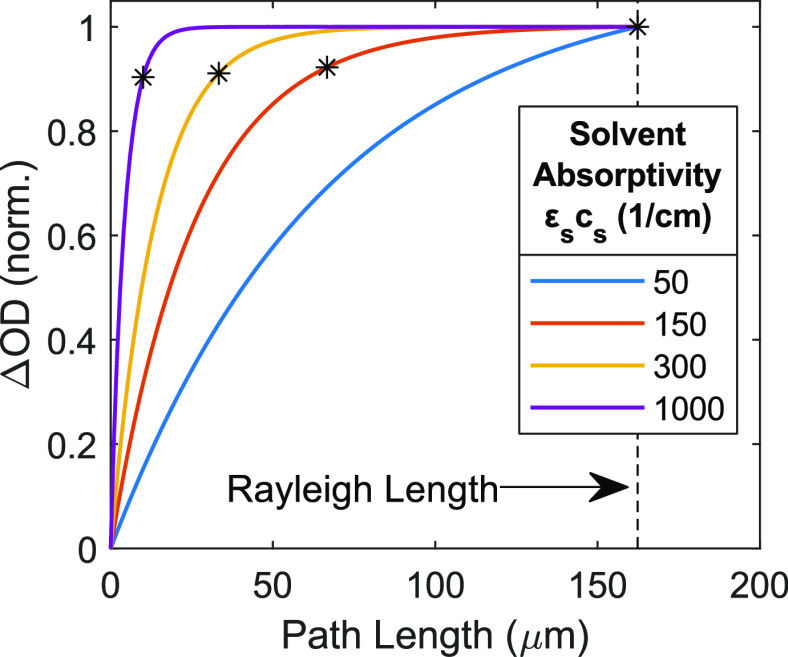
Plot of ΔOD versus path length for various solvent absorptivities. Asterisks identify points at which the path length is equal to the inverse solvent absorptivity.

The numerator of eq 6a scales bounded-exponentially with the solvent absorptivity ε_s_*c*_s_ and linearly in the denominator, the net effect approximating a hyperbolic decay. Figure 10 displays ΔOD versus path length for varying values of solvent absorptivity ε_s_*c*_s_, with ΔOD normalized to the Rayleigh length value for easier assessment. Stars denote points at which  = 1/*ε*_*s*_*c*_*s*_. The results show that signal reaches 90% or more of the asymptotic limit at path lengths equal to the inverse absorptivity of the solvent. More generally, extending path lengths longer than OD_T_ = 1 will yield diminishing gains because additional excitation is negligible beyond 90% attenuation of the pump beam. The necessity for low solvent molar absorptivity often limits the measurable frequency range for dilute analytes dissolved in water to between 2000 and 2600 cm^–1^. In that region the water bend+libration combination band peaks at 2100 cm^–1^ with ε_s_ ≈ 3.1 M^–1^ cm^–1^,^79^ corresponding to ε_s_*c*_s_ ≈ 170 cm^–1^. In that region the optimal path length for a dilute analyte would be ∼60 μm.

## 6 Enhancement by Local Oscillator Attenuation

Recognizing ΔOD as a heterodyned signal enables a clever form of signal enhancement by interference of light. From a theoretical standpoint, spectroscopists understand pump–probe signals more accurately as the third-order nonlinear response of the sample. The electric field of the third order response is *E*_sig_ ∝ χ^(3)^*I*_pump_*E*_probe_ where χ^(3)^ is the third-order susceptibility of the sample, *I*_pump_ is the intensity of the pump, and  is the electric field of the probe.^80^ At the detector, the nonlinear signal interferes with the local-oscillator field *E*_LO_ such that the total intensity is  ∝  ≈ . Then ΔOD =  =  ≈  and using , we find ΔOD in eq 14.
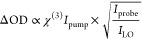
14

Therefore, accounting for local oscillator attenuation just requires multiplying ΔOD by . For the pump–probe geometry, *I*_probe_ = *I*_LO_ restricts that factor to  = 1. In four-wave-mixing, this factor is variable and enables enhancement of ΔOD when using equally strong pump and probe fields with a weak local oscillator field. However, it is possible to use this enhancement in the pump–probe geometry if one attenuates the probe *after* the sample where the probe beam then acts as the local oscillator. This strategy has been implemented in a variety of ways which work by selectively attenuating the local-oscillator field faster than the signal field after the sample, including a simple postsample polarizer,^81^ a Sagnac interferometer,^82^ or some form of attenuated reflection.^83^ In practice, the primary limitation with local-oscillator attenuation is the heterodyned signal reaches a value too small for the well depth of a detector which leads to increasing noise.

## 7 Enhancement by Plasmonics

Surface enhanced spectroscopies work by massively enhancing the electric field around the surface of small metal structures (nm to μm). The field enhancement arises from the collective oscillation of free metal electrons in response to an incident laser beam while trapped in a metal structure comparable in size to the wavelength of light. The term “plasmon”, which refers to the quantization of the oscillating electrons, stems from the observation that conduction band electrons are well-modeled as a confined gas but act as charged particles in the presence of an electric field and, hence, behave something like plasma.

Spectroscopists understand linear spectroscopy as two consecutive interactions between an oscillator and light: (1) incident light excites the oscillator, and in response, (2) the oscillator emits a “signal” that destructively interferes with the incident light, which appears as absorption.^11^ Plasmonic enhancement of linear spectra consists of four interactions: (1) incident light excites a surface plasmon on the metal structure, (2) the near-field of the surface plasmon excites the oscillator, (3) the oscillator emits a signal that interferes with the surface plasmon (known as “back action”), and (4) the surface plasmon, driven partially by the back action, emits a signal that interferes with the incident light. Though other interaction pathways among the incident light, plasmon, and oscillator do occur, the magnitude of enhanced signal from this four-step pathway is largest by at least an order of magnitude.^84^ The interaction pathway for 2D IR similarly involves a plasmon excitation and subsequent near-field interaction with the oscillator for each of the three incident fields, followed by the back action of the third-order response onto the plasmon.^85,86^ Plasmonic enhancement does come at the cost of scattering and absorption of the incident fields, and the range of enhancement only extends a few tens of nanometers from the surface into the sample.^87−89^ That being said, the enhancements in signal can be tremendous for analytes near enough to the surface.

In 2013, Donaldson and Hamm first reported a 10^2^ enhancement of 2D IR signal from thiol-functionalized gold nanoparticles.^90^ In 2015, Selig, Siffels and Rezus reported a ∼10^4^ enhancement of 2D IR signal using randomly spaced micron-long gold nanoantennas, lithographically deposited on CaF_2_.^91^ Shortly thereafter, Kraak and Hamm found that sputtering a 1–3 nm gold layer on the surface of an ATR prism enhanced the 2D IR signal of monolayers by 50–450 fold.^92,93^ Since then, additional studies have been reported on regularly spaced nanoantennas^94−97^ and trimer nanoantennas^98,99^ yielding 10^4^–10^5^ enhancement. Other studies have shown that combining plasmonic enhancement with an attenuated reflection geometry (SEAR-2D IR) enables an added mechanism for enhancement by means of local oscillator attenuation.^47,86,100,101^ In fact, Morichika et al. reported a 10^7^ enhancement of 2D IR signal by detecting the back scattering off of gold nanoantenna arrays.^47^ With the exception of Donaldson and Hamm’s gold nanoparticles, virtually all surface enhanced 2D IR experiments reported to date are of thin film samples where the surface density of chromophores is comparatively high relative to mM bulk samples. Unfortunately for bulk samples, the enhancement only extends a few tens of nanometers from the surface into the sample,^87−89^ which effectively reduces the path length of bulk measurements by a few orders of magnitude. This questions the applicability of plasmonics to conventional bulk systems, particularly given the lack of demonstration thus far.

One complication introduced by plasmonic enhancement in both linear and nonlinear measurements is a Fano-like line shape.^85,102^ The origin of this phenomenon is from two complementary phase delays: the first is acquired between the incident field that excites the plasmon and the near-field response that excites the oscillator; the second is acquired by the complement back action process.^84−86^ This effect is prominent when the size of the metal particle is comparable to the wavelength of light, such as in micron sized nanoantennas,^47,84,85,91,94,96,99,103^ but appears negligible in reports using 1–3 nm gold particles deposited by sputtering.^92,93,101^ As pointed out by Chuntonov and Rubtsov, the plasmonic Fano resonance only occurs along the probe axis and is well-modeled by classical electromagnetic theory.^85^ This can be explained by the 2D IR signal carrying a ±ϕ_1_ ∓ ϕ_2_ + ϕ_3_ phase dependence such that the phase delay cancels along the pump axis but remains along the probe axis.^85,86^ In fact, Tek and Hamm have shown how the Fano interference can be corrected by simply applying a zero-order phase shift along the probe axis.^104^

One unanswered question regarding plasmonic enhancement is whether signal enhancement comes at the cost of enhanced temperature jump of the surroundings from absorption of the pump. Since plasmonic enhancement would apply to the pump field as well, one might expect that solvents such as water should be subjected to enhanced absorption of pump light per unit volume near the surface, which would cause a larger jump in solvent temperature as discussed below in section 8. More careful measurements of surface enhanced IR spectroscopy on aqueous proteins, particularly at the high intensities required by 2D IR, are needed to quantify this effect.

## 8 Temperature Jump Consideration

Signal enhancement by higher pump fluence is an attractive strategy for weaker chromophores. However, in systems with an absorbing solvent, this presents a dilemma where enhancing signal by increasing fluence also increases the amount of light absorbed by the solvent and thereby leads to an abrupt change in temperature of the solvent surrounding the analyte. The abrupt change in temperature induced by the pump pulse(s), known as the temperature jump, manifests as fluence and time-delay dependent artifacts in pump–probe spectra as a result of temperature dependent properties such as the index of refraction, dynamics, and other line shape features more generally.^71,105^

If the absorptivity of the solvent is much larger than the analyte (ε_s_*c*_s_ ≫ ε_a_*c*_a_) and ε_s_ is approximately constant throughout the pump spectrum, e.g., water at 2100 cm^–1^, then the heat absorbed by the solvent is equal to the energy of the pump pulse times the fraction of light absorbed by the solvent, *q* = *E*_pump_(1 – ). The temperature jump, Δ*T*, following vibrational relaxation of the absorbed light is related to the specific heat capacity *C* and mass *m* of the solvent by *q* = *mC*Δ*T*. The time scale of thermal diffusion out of the excitation volume is on the order of hundreds of microseconds to milliseconds,^106^ so Δ*T* does not relax prior to the arrival of the probe pulse. Therefore, *m* = ρ*V*_ex_ where ρ is the density of the solvent and  is the pump excitation volume. These expressions combine to form an estimate of Δ*T* due to absorption of the pump light by the solvent in eq 15.
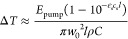
15

Comparing eqs 6a and 15, it is notable that ΔOD and Δ*T* scale identically in terms of the inverse beam waist and pump energy, hence, the trade-off between ΔOD and Δ*T* as a function of pump fluence. Equation 15 assumes a flat top beam profile and uniform excitation as a function of propagation depth. The fluence of a Gaussian beam at **r** = 0 is about ∼2× higher than the average fluence of the averaged flat top beam. Therefore, the maximum temperature jump within the sample, located at the front of the sample and center of the pump beam, is  = . It is possible to curb the temperature jump by using a pulse shaper to band narrow the pump spectrum around the analyte line shape, which reduces unnecessary absorption from the solvent. Of course, band narrowing comes with a trade-off in time resolution. As an example, consider the temperature jump of a dilute cyano oscillator in an aqueous sample with *E*_pump_ = 1 μJ, ε_s_ = 3.1 M^–1^ cm^–1^, *c*_s_ = 55 M,  = 50 μm,  = 12.5 μm, ρ = 1 g/cm^3^, and *C* = 4.186 J/g·K yields Δ*T* ≈ 8 K.

## 9 Summary

**Figure 11:**
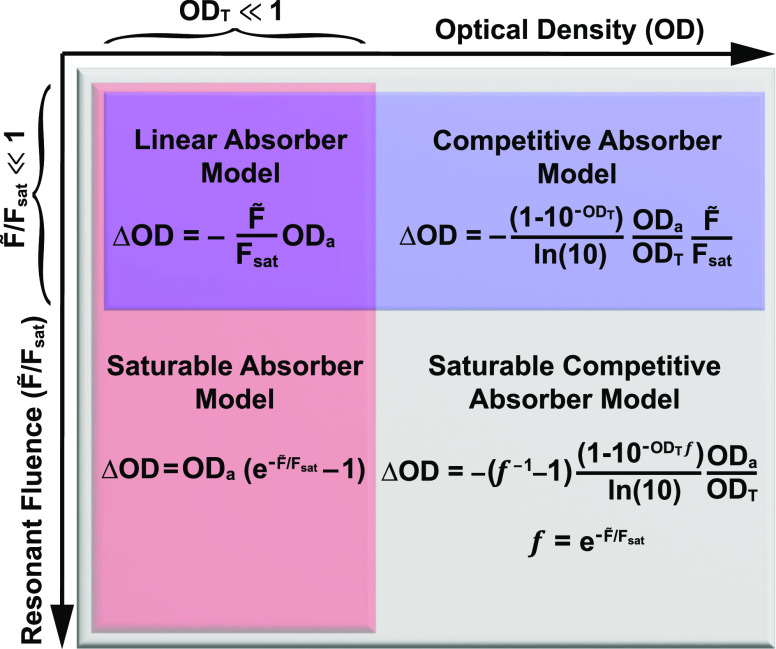
Four models of pump–probe signal under varying conditions of relative resonant fluence and total optical density. Quantities necessary to evaluate these formulas include the analyte optical density (OD_a_), total optical density (OD_T_), the fraction of pump fluence resonant with the analyte line shape (*F̃*), and the saturation fluence of the analyte (*F*_sat_).

We have reviewed several different formulas for computing the absolute magnitude of pump–probe signal over a range of conditions known to complicate signal scaling laws. Figure 11 summarizes the various regimes in which each model is valid. The saturable competitive model is suitable in every case of fluence and optical density. In the limiting case of low (total) optical density, the model simplifies to the competitive absorber model. In the limiting case of low resonant fluence, the model simplifies to the saturable absorber model. And finally, in the limiting case of both low total optical density and low resonant fluence, the model simplifies to the linear absorber model.

Evaluating these models requires knowledge of at least four experimental quantities, each of which we have reduced to some combination of the analyte molar absorptivity, concentration, path length, pulse energy, spectral bandwidth, line shape width, beam waist at focus, and the optical density of the solvent. We analyzed functional relationships between the pump–probe signal and these quantities and discussed practical limitations and trade-offs with respect to each. Several optical calculations were reviewed such as estimating the columnated range of a beam to identify the limit of propagation or the beam waist and Rayleigh length of a focused beam to estimate fluence and the maximum useful path length. We also reviewed other unconventional methods of increasing pump–probe signals, including various forms of local-oscillator attenuation and, more recently, plasmonic enhancement. Estimations of solvent heat absorption by the pump pulse suggest samples containing strongly absorbing solvents like water could have significant temperature jumps that may limit high fluence strategies including plasmonic enhancement.
